# Analysis of print news media framing of ketamine treatment in the United States and Canada from 2000 to 2015

**DOI:** 10.1371/journal.pone.0173202

**Published:** 2017-03-03

**Authors:** Melvyn W. B. Zhang, Ying X. Hong, Syeda F. Husain, Keith M. Harris, Roger C. M. Ho

**Affiliations:** 1 Center for Healthcare Innovations & Medical Engineering, National University of Singapore, Singapore; 2 Department of Psychological Medicine, Yong Loo Lin School of Medicine, National University of Singapore, Singapore; 3 School of Medicine, University of Tasmania, Hobart, TAS, Australia; 4 School of Psychology, University of Queensland, St Lucia, Qld, Australia; Chiba Daigaku, JAPAN

## Abstract

**Objectives:**

There are multifaceted views on the use of ketamine, a potentially addictive substance, to treat mental health problems. The past 15 years have seen growing media coverage of ketamine for medical and other purposes. This study examined the print news media coverage of medical and other uses of ketamine in North America to determine orientations and trends over time.

**Methods:**

Print newspaper coverage of ketamine from 2000 to 2015 was reviewed, resulting in 43 print news articles from 28 North American newspapers. A 55-item structured coding instrument was applied to assess news reports of ketamine. Items captured negative and positive aspects, therapeutic use of ketamine, and adverse side effects. Chi-squares tested for changes in trends over time.

**Results:**

In the 15-year reviewed period, the three most frequent themes related to ketamine were: abuse (68.2%), legal status (34.1%), and clinical use in anesthesia (31.8%). There was significant change in trends during two periods (2000–2007 and 2008–2015). In 2008–2015, print news media articles were significantly more likely to encourage clinical use of ketamine to treat depression (p = 0.002), to treat treatment resistant depression (p = 0.043), and to claim that ketamine is more effective than conventional antidepressants (p = 0.043).

**Conclusions:**

Our review found consistent positive changes in the portrayals of ketamine by the print news media as a therapeutic antidepressant that mirror the recent scientific publications. These changes in news media reporting might influence the popularity of ketamine use to treat clinical depression. Guidance is required for journalists on objective reporting of medical research findings, including limitations of current research evidence and potential risks of ketamine.

## Introduction

Print news media plays an important role in influencing the public perceptions of psychoactive substances. There is a strong association between media reporting and an increase in the use and misuse of psychoactive substances [1]. In the United States (US), news media framing often sets the public opinion about psychoactive substances [2]. Hence, media is expected to practice socially responsible reporting [3]. Research on news media framing provides valuable insights into the opinions of the general public on psychoactive substances, as well as the impact on society when there is a change in the classification of a psychoactive substance or governmental policies on drug control [4].

There was a recent epidemic of opioid analgesic abuse in North America, and studies on news media framing were conducted. McGinty and colleagues (2016) studied how opioid analgesic use and abuse was framed by the news media in the US from 1998 to 2012 [5]. This study revealed that news media framed the issue of opioid analgesic abuse under the criminal jurisdiction, and the most commonly proposed solutions were through legal means [5]. In contrast, another study found that the print news media in North America portrayed the abuse of another opioid analgesic, oxycodone, as a social problem, and it coincided with the reduction in oxycodone prescriptions by doctors in Nova Scotia, Canada [6]. Although news media framing research has been conducted on opioid analgesics, there is a paucity of similar research on ketamine, which is a psychoactive substance with abuse potential.

In 1962, ketamine was first synthesized by Parke-Davis laboratory, which subsequently patented ketamine as an anesthetic [7]. Ketamine is a non-competitive N-methyl-D-aspartic acid (NMDA) receptor antagonist and derived from phencyclidine (PCP), which is a hallucinogen. Ketamine binds to the PCP binding site of the NMDA receptor in the human brain. In the 1960s, ketamine was used as a battlefield anesthetic during the Vietnam War. Ketamine is a dissociative anesthetic, which is helpful in treating wounded soldiers by keeping them conscious but cognitively separated from the pain [8]. Ketamine was approved by the FDA as an anesthetic for cardiac catheterization, skin grafting, orthopedic and extraperitoneal procedures, as well as diagnostic procedures performed on the eye, ear, nose, and throat [9]. Ketamine has remained in use for the induction of general anesthesia in adult populations; however, this practice is now very uncommon owing to the side effects of ketamine, including an increase in sympathetic tone, amnesia, emergence delirium, and hallucinations. Ketamine is sometimes used as a sedative for children undergoing dental and emergency procedures because it does not cause respiratory depression and preserves the airway reflexes [10]. The intravenous route is preferred because it allows precise dosing and dose adjustment when side effects occur [11].

Ketamine, also known as “K” or “Special K,” is a psychoactive substance with abuse potential because it causes euphoria, sensory distortions, impairments in set-shifting, and heightened feelings of empathy [12]. In the US, ketamine is a Schedule III drug under the Controlled Substances Act, which limits its prescription due to its abuse liability [13]. From 1995 to 2002, emergency room visits due to ketamine abuse had increased by 20 times [14]. Ketamine is a common psychoactive substance abused by young people, and 1.5% of the US 12th graders reported ketamine abuse [15]. Ketamine abuse is associated with amnesia, dependence, and urinary incontinence [16].

Ketamine is a key compound in neurobiological research testing the glutamatergic hypothesis of psychosis and depression [17]. Several clinical trials have evaluated the slow intravenous (IV) infusion of ketamine at sub-anesthetic doses (0.5 mg/kg) and have explored its effect as a rapid antidepressant [18]. Response rates have ranged from 29% [19] to 79% [20]. Around two-third of the patients relapsed within 1 week [21] due to the short half-life of ketamine [22]. Hence, some researchers have proposed repeated infusions of ketamine [20]; however, repeated infusions may lead to detrimental side effects in the brain [23]. The main difference between the application in clinical trials and illicit use of ketamine is the dosage and frequency of administration [24], as well as route of administration [7]. In clinical trials, the average dose is a single infusion of 35 mg for 5–7 days; however, illicit users may take several grams of ketamine per day by smoking, insufflation, or intramuscular injection. Furthermore, clinical trials do not provide evidence of the real-world experience to predict how patients with depression interact with ketamine outside the rigors of a clinical trial [24, 25]. Rasmussen (2015) emphasized that the safety of prolonged ketamine use in clinical settings has not been established, and therapeutic enthusiasm must be tempered by its addiction liability [26]. Ho and Zhang (2016) performed a critical appraisal of the clinical trials that evaluated ketamine as an antidepressant [7]. The results should be interpreted with caution due to the following reasons. First, most of these trials compared ketamine with placebo. There was a lack of direct comparisons between ketamine and other antidepressants in most trials [11]. Second, the follow-up period was too short to rule out addiction and complications associated with regular ketamine infusion. Third, ketamine was used with other psychotropic medications in some clinical trials [19]. Fourth, the aforementioned studies were not able to ascertain the differences between the rapid antidepressant effect of ketamine and the “drug high” of psychoactive substances. Ketamine use for treating severe depression leads to major ethical concerns including the lack of safety data in off-label use and its abuse potential [24].

Due to the growing number of ketamine trials, print news media has conducted interviews with researchers and reported their findings. A 2016 Washington Post article was headlined “One-time party drug (ketamine) hailed as a miracle for treating severe depression” [27]. In this article, ketamine was described as the “next big thing” in psychiatry with rapid effect and booster treatment. After reading this article, patients may request their doctors to prescribe ketamine that is described as a miracle anti-depressant. Cautious clinicians and academics are concerned that the benefits of ketamine could be exaggerated by print media articles [24, 28, 29]. The aforementioned article is very different from a 2003 Portsmouth Herald article with the headline “Parents enlisted drug battle.” In this article, ketamine was described as special K or a horse tranquilizer that produced superhuman strength, lysergic acid diethylamide (LSD)-like hallucinations, amnesia, depression, and long-term memory loss [30]. Given the changes in the uses of ketamine across the years, as well as the recent media promotion of ketamine as a rapid antidepressant, it is pertinent to evaluate any changes in the trends of how print news media reports on ketamine. To our knowledge, no studies have examined the print news media coverage of ketamine. Our objective was to examine how ketamine had been portrayed by print news media framing research analysis. The results of this study have important implications for media, medical professionals, and patients.

## Methodology

This study followed the systematic and quantitative approach proposed by McGinty et al. (2016) for their analyses of how news media frame opioid analgesic abuse in the US [5]. For this study, we focused on the characteristics and changes in the trends of reporting on ketamine by print news articles. The study examined English language print news media articles in the US and Canada related to ketamine that were published from 2000 to 2015. News sources included all newspaper articles of at least 100 words published and circulated in the US and Canada. We used NewspaperARCHIVE.com, a newspaper archive website that has been widely used by tertiary educational institutes and contains the largest number of archived newspaper articles published in the US and Canada since 1607 [31]. The online database was searched for articles published from 2000 to 2015. We used “ketamine” as the main search term to identify articles discussing other uses of ketamine. Then, we refined our search by using the following combinations of search terms based on another media framing study on opioid use and abuse [5]: ketamine AND “abuse,” ketamine AND “addict,” ketamine AND “depress*,” ketamine AND “mood,” ketamine AND “misuse,” and ketamine AND “overdose.” The search yielded a cumulative total of 112 newspaper articles. The newspaper articles were retrieved online. The articles were assessed by the authors (YXH and MWZ), and 43 articles were included in the final analyses.

The following information was extracted from each news article by the first author (MWBZ) and cross-examined by the second (YXH) and last author (RCMH). A 55-item standardized data collection form based on the prior protocols for review was applied to study the characteristics of print media articles on ketamine, and the following information was recorded: a) Ketamine as a drug of abuse, b) The mechanisms of action of ketamine, c) Clinical applications of ketamine, d) Legal status and ethical issues associated with ketamine usage, e) Overall theme of the news article, e) Negative aspects of ketamine as a potential antidepressant, f) Positive aspects of ketamine as a potential antidepressant, and g) Adverse consequences of ketamine usage. For each of the individual domains, specific questions were devised to assess the data reported in the news articles comprehensively.

*Ketamine as a drug of abuse*: We assessed whether the news articles mentioned ketamine as a derivative of a hallucinogen, a controlled drug, its addiction potential, the harmful effects of regular ketamine use, the prevalence of ketamine abuse in the general community, and other forms of misuse.

*Mechanism of action of ketamine*: We assessed whether the news articles described the mechanism of action of ketamine and other mechanisms of action.

*Clinical Applications of ketamine*: We measured whether the news articles described the clinical applications of ketamine in anesthesia, as a therapeutic treatment for depressive disorders, bipolar disorder, and chronic pain disorders (such as reflex sympathetic dystrophy).

*Legal status and ethical issues*: We assessed whether the news articles described the schedule and legal status of ketamine, arrest and prosecution of ketamine abusers, financial conflicts of the interest of researchers or pharmaceutical companies, abuse of ketamine by health professionals, inappropriate prescription of ketamine by doctors, and other legal aspects not mentioned above.

*Overall theme of the news articles*: We assessed the articles and classified them in the following themes: 1) Coverage of the illegal aspects of ketamine use, 2) Coverage of the proper use and danger of ketamine, 3) Usage of ketamine to treat medical disorders, 4) Usage of ketamine to treat psychiatric disorders and other themes not mentioned above.

*Adverse effects of ketamine*: We assessed whether the articles mentioned the side effects associated with ketamine. In particular, neuropsychiatric side effects, cardiovascular side effects, urinary side effects, and liver side effects were categorized. Morbidity and mortality due to the usage of ketamine were also recorded.

### Data analysis

After the initial screening of the print news media articles, the information extracted from the articles were coded for further analyses. The proportion of studies that mentioned each of the aforementioned domains were computed and compiled into the respective tables. Chi-square tests were used to determine whether the changes in the trends over time were of statistical significance. A p value of less than 0.05 was set as the level of statistical significance. Statistical analyses were conducted using Social Package for Social Sciences (SPSS) v. 23 (IBM Corp.).

## Results

A total of 112 articles were identified using the search strategy, 37 articles were eliminated because the lengths were shorter than 100 words or could not provide adequate information for the 55-item standardized data collection. Thirty-two articles were eliminated because they were duplicate publications on the same day. Finally, 43 articles were included for analyses. There were 21 articles published in 2000–2007 and 22 articles published in 2008–2015.

Table 1 summarizes the characteristics mentioned by the news articles (n = 43). The three most frequent themes included the following: 1) Issues of ketamine abuse or illegal aspects of ketamine use (68.2%), 2) The schedule and legal status of ketamine (34.1%), and 3) The clinical use of ketamine in anesthesia (31.8%). None of the articles mentioned the abuse of ketamine by health professionals or inappropriate prescriptions of ketamine by doctors. Twelve news articles (27.3%) reported that ketamine could be used for treating depressive disorders. Approximately, 22.7% of the news articles reported the addiction potential of ketamine, and 20.5% reported that ketamine is a controlled drug.

**Table 1:** Characteristics of ketamine described by print news media articles from 2000 to 2015.

Characteristics mentioned by print news media articles (n = 43)	N	Pct.
**Ketamine as a drug of abuse**		
A derivative of a hallucinogen, phencyclidine (PCP).	5	11.4%
A controlled drug.	9	20.5%
Has addiction potential.	10	22.7%
Harmful effects of regular use	8	18.2%
Prevalence of abuse in the community	10	22.7%
• Date rape drug	6	13.6%
• Club drug	13	29.5%
• Others	6	13.6%
**The mechanisms of action**		
N-methyl-D-aspartic acid (NMDA) receptor antagonist	2	4.5%
Other mechanisms (e.g. PCP binding site, dopaminergic, opioid effects, release of glutamate and potential neurotoxicity)	2	4.5%
**Clinical applications**		
Anesthesia	14	31.8%
Depressive disorder	12	27.3%
Bipolar disorder	1	2.3%
Chronic pain e.g. reflex sympathetic dystrophy (RSD)	3	6.8%
• Veterinary tranquiliser	13	29.5%
• Veterinary aesthetic	4	9.1%
**Legal and ethical issues**		
Schedule and legal status	15	34.1%
Arrests and prosecutions	6	13.6%
Financial conflicts of interest	3	6.8%
Stealing of ketamine from veterinary doctors	1	2.3%
**Overall theme of news article**		
Ketamine abuse or illegal aspects	30	68.2%
Educational and informs public on proper uses and dangers	13	29.5%
Promotes ketamine to treat medical disorders	6	13.6%
Promotes ketamine to treat psychiatric disorders	10	22.7%
• Date rape drug warnings	3	6.8%
• Sponsored articles by pharmaceutical companies	4	9.1%
• Public education (other)	7	15.9%
• Legal aspects	3	6.8%
• Clinical trials/alternative clinical uses	6	13.6%

Table 2 illustrates the changes in the trends of reporting the negative and positive aspects of ketamine as a potential antidepressant over the study years. For the negative aspects of ketamine as a potential antidepressant, there were no major changes in the trends from 2000–2007 to 2008–2015 (all p > 0.05). For the positive aspects of ketamine as a potential antidepressant, the most frequently mentioned positive aspects with statistically significant changes were the following: the article encouraged the clinical use of ketamine to treat clinical depression (20.5% in 2008–2015 vs. 2.3% in 2000–2007, p = 0.002), ketamine can treat treatment-resistant depression (15.9% in 2008–2015 vs. 4.5% in 2000–2007, p = 0.043), and ketamine was more effective than conventional antidepressants (15.9% in 2008–2015 vs. 4.5% in 2000–2007, p = 0.043). Two items approached statistical significance, ketamine as a novel antidepressant (18.2% in 2008–2015 vs. 6.8% in 2000–2007, p = 0.055) and positive claims of ketamine were based on research findings (18.2% in 2008–2015 vs. 6.8% in 2000–2007, p = 0.055).

**Table 2:** Changes over time in reporting negative and positive aspects of ketamine as a potential antidepressant.

	2000–2007		2008–2015		Change over time	
	(21 articles)		(22 articles)		Change over time	
Trends	N	Pct.	N	Pct.	Trend	P
**Negative aspects**						
Can be addictive	0	0.0%	2	4.5%	Increasing	0.130
Serious side effects	0	0.0%	1	2.3%	Increasing	0.290
Antidepressant effect short-lived	1	2.3%	2	4.5%	Increasing	0.496
Can crash to depression after administration	0	0.0%	1	2.3%	Increasing	0.290
Negative research findings	0	0.0%	1	2.3%	Increasing	0.290
Other negative aspects	0	0.0%	1	2.3%	Increasing	0.290
**Positive aspects**						
A “novel” antidepressant	3	6.8%	8	18.2%	Increasing	0.055
High response rate	2	4.5%	4	9.1%	Increasing	0.318
Treatment resistant depression effectiveness	2	4.5%	7	15.9%	Increasing	0.043
Can prevent suicide	2	4.5%	4	9.1%	Increasing	0.318
More effective than placebo	1	2.3%	2	4.5%	Increasing	0.496
More effective than conventional treatment	2	4.5%	7	15.9%	Increasing	0.043
More convenient routes of administration	0	0.0%	2	4.5%	Increasing	0.130
Positive research findings	3	6.8%	8	18.2%	Increasing	0.055
Other positive aspects	2	4.5%	3	6.8%	Increasing	0.118
**Conventional and alternative antidepressant treatment**						
Effectiveness of current treatment	2	4.5	5	11.4%	Increasing	0.171
Effectiveness of psychotherapy	0	0.0%	1	2.3%	Increasing	0.290
**Overall theme**						
Discourages clinical use to treat depression	0	0.0%	1	2.3%	Increasing	0.290
Encourages clinical use to treat depression	1	2.3%	9	20.5%	Increasing	0.002
Neutral on clinical use to treat depression	1	2.3%	0	0.0%	Decreasing	0.334

Increasing = trends show these factors are increasing relative to earlier reports. Decreasing = trends show these factors are decreasing relative to earlier reports. P values refer to chi-square tests between the two time periods.

Table 3 summarizes the adverse effects of ketamine reported by media articles. For neuropsychiatric side effects, the most commonly reported side effects were perceptual disturbances and psychosis (38.6%), followed by memory loss (25.0%) and dissociative experiences (25.0%). Additionally, cardiovascular side effects (11.4%), serious side effects associated with ketamine overdoses and complications (20.5%), and deaths (9.1%) were reported.

**Table 3:** Adverse effects of ketamine described by print news media articles, 2000–2015.

Adverse effects	N	Pct.
**Neuropsychiatric side effects**		
Aggression	1	2.3%
Confusion	6	13.6%
Dissociation	11	25.0%
Memory loss	11	25.0%
Perceptual disturbances/psychosis	17	38.6%
Vivid dreams	1	2.3%
Slurred speech	3	6.8%
**Cardiovascular side effects**		
Increase in blood pressure	5	11.4%
**Renal side effects**		
Urinary incontinence	1	2.3
**Liver side effects**		
Impaired liver function	0	0.0%
**Serious side effects**		
Ketamine overdoses and complications	9	20.5%
Death	4	9.1%
Other side effects (e.g. depression, dream-like state, euphoria, loss of consciousness, long term cognitive impairments, poor attention, respiratory depression)	14	31.8%

## Discussion

To our knowledge, this is the first media framing study to compare how the print news media described ketamine during two periods (2000–2007 and 2008–2015). Our analyses showed that newspaper articles published in 2008–2015 were more likely to report that ketamine treatment was more effective than conventional antidepressants and were more encouraging of ketamine clinical use to treat depression as compared to the period 2000–2007. It is not surprising that very few print news articles mentioned the therapeutic benefits of ketamine from 2000 to 2007 as, for that period, emergency visits related to ketamine misuse increased by 2,000% in the US [14]. It is possible that few print news articles reported this prior to 2007 because the majority of trials and clinical evidence in this area was only published after 2007.

In this study, we observed that the upward trend of making positive claims of ketamine based on research data by news articles was approaching statistical significance. Researchers from different countries are competing to replicate the rapid antidepressant effects of ketamine [7]. They often present their findings as a breakthrough and describe the effect of ketamine as a novel antidepressant. A British newspaper, *The Guardian*, published the results of the first UK study to administer ketamine to patients with severe depression based on a press release from Oxford University. Notably, the Oxford trial was based on a small case series of 28 patients and included flaws in study design such as the lack of placebo-control, concurrent use and increasing doses of other psychotropic medications, and the assessment of suicidal ideation based on a single question [19]. Hence, the *British Medical Journal* published an article emphasizing that the news media should not overplay the effect of ketamine but also report the risks of abuse and long-term complications [27].

There was significant increase in percentage of news articles that encourage clinical use to treat depression from 2.3% in 2000–2007 to 20.5% in 2008–2015. These articles might encourage patients with depression to request for ketamine treatment. Patients with severe depression may not have the full mental capacity for consenting to ketamine treatment [32]. Further, patients may not have an adequate understanding of the potential harm associated with long-term ketamine use [33]. Some researchers made a distinction between the application of ketamine in clinical trials and its illicit use in terms of the dosage and frequency [24]. Such distinction will be unclear if patients are allowed to administer ketamine on their own by intranasal formulation [7]. Furthermore, clinicians may also feel pressured to prescribe ketamine to treat depression, especially by patients who are abusing ketamine and hiding such histories [7]. Some clinicians are concerned about the potential diversion [24] and doctor-shopping from different commercial clinics to obtain high doses of ketamine [32]. This concern is relevant in North America because Americans were found to be less likely to face legal consequences for the misuse of prescription medication than the misuse of illicit drugs [3]. Healthcare professionals who have access to ketamine may also develop an addiction to ketamine following repeated self-administrations [34]. Off-label ketamine use in treating depressive disorders may breach ethical and moral standards, especially in communities seriously affected by ketamine abuse [33].

In this print news media framing study on ketamine, we found that news articles were significantly more likely to describe ketamine as more effective than conventional antidepressants in 2008–2015. Such arguments are not well supported by empirical evidence on the nature of depressive disorders. Most of the recent trials on ketamine did not compare the efficacy of ketamine with other standard antidepressants [29]. The Cochrane Collaboration conducted a systematic review of previous ketamine trials and concluded that there was a limited superiority of ketamine over placebos in treating mood disorders [35, 36]. Hence, ketamine was not recommended to be used as an antidepressant. Some previous studies did not use ketamine as a sole agent but administered other psychotropic medications concurrently [19, 37]. The current response rate to conventional antidepressants is from 62% to 67% [38], and the mean response rate of ketamine is around 54% (ranging from 29% to 79%) [33]. Hence, the Canadian Agency for Drugs and Technologies in Health concluded that there is a lack of evidence to recommend ketamine for treating depressive disorders [11]. In addition, a large number of patients were undertreated by conventional antidepressants and resulted in pseudo-resistance to treatment [39]. Environmental factors, including unemployment, divorce, and social isolation, also play important roles in the etiology of depressive disorders [7]. Ketamine has not been found to be better than conventional antidepressants to address these environmental factors. Treatment for severe depressive disorders should integrate biological and psychological therapies [7].

Due to its abuse liability and adverse effects, ketamine is unlikely to be widely used to treat depressive disorders in the near future. [40]. Ketamine is a racemic mixture of (S)-ketamine and (R)–ketamine [41]. The R-stereoisomer has less psychotomimetic side effects [42]. Researchers identified hydroxynorketamine, a major metabolite of R-stereoisomer, which shows an antidepressant effect and may be a safer antidepressant [41]. Media should state that the current findings are preliminary and further research needs to be performed before reaching a conclusion. In this study, the upward trend of describing ketamine as a “novel” antidepressant by print news media articles was approaching statistical significance. Newport et al. and the American Psychiatric Association (APA) Council of Research Task Force on Novel Biomarkers and Treatments cautioned against using ketamine as a novel antidepressant [43]. The rapid antidepressant effect of ketamine is not novel because most psychoactive substances, including amphetamine or cocaine, demonstrate rapid mood elevation effects [7]. The rapid mood elevation effects associated with psychoactive substances are short-lived, and users crash back to depressive states after stopping the use [44]. Users try to avoid crashes in the mood by taking more of the psychoactive substances, and this leads to addiction [7]. Patients with depression who experience a dramatic beneficial response to ketamine may face a serious fall in morale following rapid relapse [24]. In contrast, conventional antidepressants are often associated with improvement in depressive symptoms by the end of the first week of use [45].

The APA Council of Research Task Force on Novel Treatments warned that repeated administration of ketamine would raise safety concerns as compared to one-off usage when ketamine is used as a general anesthetic [43]. Prolonged usage of ketamine may lead to neurotoxicity, cognitive dysfunction [46], psychotomimetic effects, cardiovascular events, and uropathic effects [47]. The doses that cause toxicity vary among therapeutic doses (0.23–0.5 mg/kg) causing impairment of executive function [48], 2 mg/kg causing worsening cerebral atrophy [49], and very high doses (18.5 g/week) causing uropathy [50]. In this study, we found that the concerns raised by professional bodies, such as the APA, were rarely mentioned in the news articles. News articles should present the risks of ketamine; however, only 4.5% and 2.3% of the news articles published from 2000 to 2015 mentioned the addiction potential of ketamine or serious side effects, respectively. Some ketamine clinics are run by psychiatrists in the US who charge patients USD 200 to 500 per ketamine infusion to treat depression [51]. None of the print news articles reported the potential danger of administering ketamine by doctors without adequate training in anesthesia or contraindications that make patients unsuitable for ketamine. The print news media articles mainly covered the acute side effects such as psychosis, perceptual disturbances, and increase in blood pressure; they should also state the long-term side effects that include worsening depression and urinary incontinence [52]. It is a concern that only one news article reported urinary incontinence and journalists under-reported this important side effect.

Our study is the first to examine the changes in the trends of how print news media articles reported ketamine. This study has several strengths. First, we acquired news articles from a diverse range of local newspapers circulated in the US and Canada (Table 4). Second, we compared the trends of how print news media reported on ketamine over a cumulative period of 15 years. Third, we developed the pre-study assessment criteria and adopted a systematic method for data extraction and content analysis. There are also inherent limitations in this study. First, this study was focused on print news media articles published in the US and Canada and is not generalizable to other countries. Second, we did not capture and analyze articles from other forms of sources of media such as the internet, radio, and television.

**Table 4:** Newspapers included in this study.

American Newspapers	Canadian Newspapers
**South**	**Midwest**
Brazosport Facts (2 articles)	Lethbridge Herald (1 article)
Cumberland Times News (2 articles)	Winnipeg Free Press (1 article)
Galveston County Daily News (2 articles)	
Gastonia Gaston Gazette (1 article)	
Nashua Telegraph (1 article)	
**Southwest**	
Rosewell Daily Record (1 article)	
**West**	
Colorado Springs Gazette (3 articles)	
Annapolis Capital (1 article)	
**Northeast**	
Bedford Gazette (2 articles)	
Doylestown Intelligencer (2 articles)	
Gettysburg Times (1 article)	
Fitchburg Sentinel and Enterprise (1 article)	
New Castle News (1 article)	
Portsmouth Herald (1 article)	
Syracuse Post Standard (1 article)	
Tribune Democrat (1 article)	
ictorville Daily Press (1 article)	
Victorville Press Dispatch (1 article)	
**Midwest**	
Burlington Hawk Eye (1 article)	
Cedar Rapids Gazette (3 articles)	
Daily Herald Suburban Chicago (5 articles)	
Elyria Chronicle Telegram (1 article)	
Hutchinson News (3 articles)	
Indiana Gazette (1 article)	
Joplin Globe (1 article)	
Logansport Pharos Tribune (1 article)	

## Conclusion

This study is the first print news media framing analysis that examined the changes in the patterns of reporting ketamine (covering years 2000–2007 and 2008–2015). In recent years, news articles were significantly more likely to encourage the clinical use of ketamine to address treatment-resistant depression and described ketamine as more effective than conventional antidepressant treatments. The changes in patterns of reporting ketamine by media were parallel with the recent findings reported by scientific literature on ketamine. Hence, the medical communities should be more critical of the evidence provided by ketamine trials; furthermore, printed media should emphasize that use of ketamine as an antidepressant is limited. Formal guidance is required to assist journalists with how to report the potential risks of psychoactive substances such as ketamine and not devalue the evidence supporting conventional antidepressant treatments.

## Supporting information

S1 Fig Flow Chart to illustrate search strategy. (DOCX)

S1 Data Data File. (XLSX)
