# Supplementary material for: Analysis of print news media framing of ketamine treatment in the United States and Canada from 2000 to 2015
Source: PLoS One. 2017 Mar 3;12(3):e0173202. doi: 10.1371/journal.pone.0173202 (PMC5336274; doi:10.1371/journal.pone.0173202)
Supplement: S1 Fig — (DOCX) [file pone.0173202.s001.docx]

**Appendix 1 Flow chart to illustrate search strategy**

**Search strategy:**

The word “ketamine” was used as the main search term to identify articles discussing other uses of ketamine.

The following combinations of search terms were also used: ketamine AND “abuse”, ketamine AND “addict”, ketamine AND “depress*”, ketamine AND “mood”, ketamine AND “misuse” and ketamine AND “overdose”.

*truncated

The search yielded a cumulative total of112 newspaper articles.

37 articles were eliminated because the length was shorter than 100 words or could not provide adequate information for the 55-item standardized data collection.

32 articles were eliminated as they were duplicate publications on the same day.

43 articles were included for analyses.
